# Does air pollution play a role in infertility?: a systematic review

**DOI:** 10.1186/s12940-017-0291-8

**Published:** 2017-07-28

**Authors:** Julie Carré, Nicolas Gatimel, Jessika Moreau, Jean Parinaud, Roger Léandri

**Affiliations:** 1 Médecine de la Reproduction, CHU Toulouse, 31059 Toulouse, France; 2 Groupe de Recherche en Fertilité Humaine EA 3694, Université Paul Sabatier, 31059 Toulouse, France; 3 Médecine de la Reproduction, CHU Paule de Viguier, 330 avenue de Grande Bretagne, 31059 Toulouse, France

**Keywords:** Fertility, Air quality, Pollutants, Reproduction, Infertility

## Abstract

**Background:**

Air pollution is involved in many pathologies. These pollutants act through several mechanisms that can affect numerous physiological functions, including reproduction: as endocrine disruptors or reactive oxygen species inducers, and through the formation of DNA adducts and/or epigenetic modifications. We conducted a systematic review of the published literature on the impact of air pollution on reproductive function.

Eligible studies were selected from an electronic literature search from the PUBMED database from January 2000 to February 2016 and associated references in published studies. Search terms included (1) ovary or follicle or oocyte or testis or testicular or sperm or spermatozoa or fertility or infertility and (2) air quality or O_3_ or NO_2_ or PM2.5 or diesel or SO_2_ or traffic or PM10 or air pollution or air pollutants. The literature search was conducted in accordance with the Preferred Reporting Items for Systematic Reviews and Meta-Analyses (PRISMA) guidelines. We have included the human and animal studies corresponding to the search terms and published in English. We have excluded articles whose results did not concern fertility or gamete function and those focused on cancer or allergy. We have also excluded genetic, auto-immune or iatrogenic causes of reduced reproduction function from our analysis. Finally, we have excluded animal data that does not concern mammals and studies based on results from in vitro culture. Data have been grouped according to the studied pollutants in order to synthetize their impact on fertility and the molecular pathways involved.

**Conclusion:**

Both animal and human epidemiological studies support the idea that air pollutants cause defects during gametogenesis leading to a drop in reproductive capacities in exposed populations. Air quality has an impact on overall health as well as on the reproductive function, so increased awareness of environmental protection issues is needed among the general public and the authorities.

## Introduction

For decades, a causal relationship has been suspected between air pollution and some human health problems. Particulate matter (PM) and ground-level ozone (O_3_) are Europe’s most problematic pollutants in terms of harm to human health, followed by benzo (a) pyrene (BaP) (an indicator for polycyclic aromatic hydrocarbons (PAHs)) and nitrogen dioxide (NO_2_) [1]. The main sources of these pollutants are transport and energy followed by industry. Air pollution is involved in cardiovascular disease [2], stroke [3] and respiratory diseases [4, 5] such as lung cancer [6], childhood asthma [7] and atopic dermatitis [8]. Furthermore, perinatal exposure to polycyclic aromatic hydrocarbons (PAHs), nitrogen dioxide (NO_2_) and particulate matter (PM) has been demonstrated to have a negative impact on neuropsychological development in children [9]. A study carried out in Sydney estimated that reducing particulate matter (PM2.5) by 10% for 10 years would avoid approximately 650 premature deaths [10]. According to Lelieveld et al., model projections based on a business-as-usual emission scenario indicate that the contribution of outdoor air pollution to premature mortality could double by 2050 [11].

Several mechanisms of action may be involved in these health impacts: (1) Endocrine disruptor activity: This is the case of the PAHs and heavy metals (Cu, Pb, Zn, etc.) contained in particulate matter, especially from diesel exhaust [12–15]. Diesel exhaust particles contain for example substances with estrogenic, antiestrogenic and antiandrogenic activities that can affect gonadal steroidogenesis and gametogenesis. (2) Generation of oxidative stress: NO_2_, O_3_ or PM (through the heavy metals and PAHs they contain) can generate reactive oxygen species (ROS) [16–18]. These cause alterations in DNA, proteins and membrane lipids [19, 20]. (3) Modifications of DNA: Through the formation of DNA adducts, leading to modifications in gene expression and/or the appearance of epigenetic mutations or modifications such as an alteration of DNA methylation [21–23].

These are general mechanisms that can affect all functions, including procreation.

The purpose of this study was to evaluate our current understanding of air pollution’s impact on reproductive functions. The results of such a review could sensitize the population and the authorities to take care of air quality.

## Methods

### Research strategy

We have conducted a systematic review of the literature concerning the exposure to environmental air pollutants and fertility or reproductive health. This analysis was made in compliance with the PRISMA criteria: Preferred Reporting Items for Systematic Reviews and Meta-Analyses [24]. We conducted the research in April 2016 using the PubMed database. All of the research was done using the Advanced Search Builder and the key words were searched in [Title OR Abstract]. Over the period from 01/01/2000 and 04/01/2016, we filtered the hits by selecting articles written in English.

Regarding fertility, we used comprehensive terms in order to optimize the search. Regarding the air quality, we opted for a strategy that combined comprehensive terms and pollutant names so as not to omit any articles.

Therefore, the search was as follows:

(((((((((((((((((air quality [Title/Abstract]) OR O3[Title/Abstract]) OR NO2 [Title/Abstract]) OR Ozone[Title/Abstract]) OR PM2.5 [Title/Abstract]) OR diesel [Title/Abstract]) OR SO2[Title/Abstract]) OR traffic[Title/Abstract])) OR PM10 [Title/Abstract])) OR air pollution[Title/Abstract]) OR air pollutants [Title/Abstract]) AND (“2000/01/01” [PDat]: “2016/04/01” [PDat]))) AND ((((((((((((ovary [Title/Abstract]) OR follicle*[Title/Abstract]) OR follicular [Title/Abstract]) OR ovaries [Title/Abstract]) OR oocyte*[Title/Abstract]) OR testis [Title/Abstract]) OR testicular [Title/Abstract]) OR sperm [Title/Abstract]) OR spermatozoa [Title/Abstract]) OR fertility [Title/Abstract]) OR infertility [Title/Abstract]) AND (“2000/01/01” [PDat]: “2016/04/01” [PDat]))) NOT ((((Cancer [Title/Abstract]) OR allergy [Title/Abstract]) OR bacteria [Title/Abstract]) AND (“2000/01/01” [PDat]: “2016/04/01” [PDat]))) AND “english” [Language].

### Article inclusion and exclusion criteria

We have included all of the human and animal studies arising from the search.

**Fig. 1:**
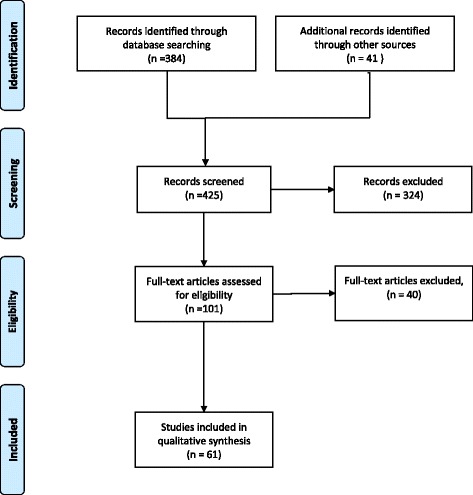
Flow chart describing the selection of articles

We have excluded articles that included results that did not concern fertility, those focused on ovarian cancer, polycystic ovary syndrome, endometriosis or precocious puberty. Finally, we have excluded animal data that do not concern mammals and studies based on results from in vitro culture (Fig. 1). However, non-mammal and in vitro studies were included for informing the mechanisms of pollutants’ action.

## Results

### **Impact of air pollution on spontaneous fertility** (Table 1)

Few studies carried out on animals suggest a negative impact of exposure to air pollution on spontaneous fertility [25, 26]. Two studies were carried out on mice in the city of Sao Paulo, Brazil, which has a high level of air pollution. Mohallem et al. found a significant reduction in the number of newborns per mouse and a significant increase in the embryo implantation failure rate in female mice exposed as newborns for 3 months to the city polluted air and then mated with non-exposed males as adults [27]. No effect was evidenced when exposure occurred during adulthood. Veras et al., on the other hand, reported a significant increased number of days in estrus over the studied period (mean (SD): 56.63 (11.65) vs 34.57 (6.68); *p* < 0.03), a reduction in the number of ovarian antral follicles (mean (SD): 75 (35.2) vs 118.6 (18.4); *p* < 0.04), an increase in the time to mating as well as a significant decrease in the fertility index (number of pregnant females/total number of females, Table 1) in adult mice exposed to pollution from automobile traffic [28].

**Table 1:** Effect of air pollutants on spontaneous fertility

Publication	Species	Number of subjects	Air pollutant (s) studied	Methodology	Results
Mohallem et al., 2005 [27]	Mice	104	Multiple ambient pollutants from automobile traffic	Mice divided into 4 groups maintained in chambers at least 3 months: - Group 1: adults (aged 10 weeks, *n* = 20) exposed to filtered air - Group 2: adults (*n* = 20) exposed to ambient air pollutants - Group 3: newborns (aged 10 days, *n* = 33) exposed to filtered air - Group 4: newborns (*n* = 31) exposed to ambient air pollutants	No effects of exposure in adulthood. In group 4 compared to group 3, decreased number of newborns per mouse (mean ± range: 4.0 ± 6.0 vs 6.0 ± 7.0; *p*< 0.04) and increased embryo implantation failure rate (3.5 ± 7.0 vs 2.0 ± 8.0; *p* < 0.05).
Veras et al., 2009 [28]	Mice	60	Multiple ambient pollutants from automobile traffic	Second-generation mice (age > 60 days) born of couples raised in chamber with filtered air (F) or non-filtered air (NF), divided into 2 categories: - 10 females F and 10 NF used for assessing cycles and ovarian follicles - 40 mated mice divided into 4 groups: - F1: mice raised in F with pregnancy in F - F2: mice raised in F with pregnancy in NF - NF1: mice raised in NF with pregnancy in F - NF2: mice raised in NF with pregnancy in NF	In NF mice compared to F mice: Increased time to mating (mean days (SD): 10.65 (5.77) vs 3.5 (1.54); *p* < 0.0001) Decreased fertility index (number of pregnant females/total number of females: 55 vs 95%; *p* < 0.003). Increase in spontaneous abortion rate in NF2 group compared to F1 (mean (SD): 41.7 (5.8) vs 12.1 (5.8); *p* < 0.005).
Dejmek et al., 2000 [29]	Humans	2585 couples	SO_2_	Retrospective birth cohort study: Measured average monthly exposure of couples during 4 months before 1st cycle with unprotected intercourse (based on estimated date of conception).	Compared with the reference SO_2_ exposure level (<40 pg/m^3^): Adjusted Odd Ratio for fecundability rate: 0.57 (CI, 0.37–0.88) for medium level exposure (40–80 pg/m^3^): 0.49 (CI 0.29–0.8 1) for high level exposure (>80 pg/m^3^):
Slama et al., 2013 [30]	Humans	1916 couples	SO_2_, NO_2_, PM2.5, O_3,_ PAH	Retrospective birth cohort study: Measured average exposure of couples during 2 first months prior to 1st cycle with unprotected intercourse.	Decrease adjusted fecundability ratio (FR [95%CI]) with PM2.5 (0.78 [0.65–0.94]) and NO_2_ levels (0.72 [0.53–0.97]).
Nieuwenhuijsen et al., 2014 [31]	Humans	All women of reproductive age (15–44 years) living in Barcelona: mean (SD) 368.92 (±111.22) women per census tract (*N* = 1061 census tracts)	PM10, PM2.5–10, PM2.5, NO_2_, NOx	Cross sectional study: Measured average level of exposure of women of reproductive age (15–44 years) living in Barcelona, according to census tract of residence.	Risk ratio [95% CI] for reduced fertility rate (number of live births per 1000 women) = 0.87 [0.82–0.94] per interquartile range of PM2.5–10.
Mahalingaiah et al., 2016 [32]	Humans	36,294 nurses of reproductive age	Living in proximity to major roads; PM10, PM2.5–10, PM2.5	Prospective cohort study: Biannual questionnaire on fertility Residential address proximity to major roads (2 categories: 0–199 m and ≥200 m). Predicted ambient exposure to PM10, PM2.5–10 and PM2.5 at residential address	Hazard Ratio [95% CI] for infertility: 1.11 (CI: 1.02–1.20) for living close to major roads and 1.10 (0.99–1.22) for each 10 mg/m^3^ increase in cumulative average exposure to PM2.5–10.

Studies carried out on humans in different countries have produced concordant results regarding an impact of polluted air on human fertility although they are discordant regarding the type of air pollutant concerned. In Teplice, a highly polluted district in Czech Republic, Dejmek and colleagues have studied the effect of SO_2_ exposure during the 4 previous months before conception in a birth cohort of 2585 parental pairs. They found a significantly negative impact of sulfur oxide (SO_2_) exposure in the second month before conception on fecundability rate (assessed as the pregnancy rates after the 1st menstrual cycle without contraception): the adjusted odd ratios were 0.57 (CI, 0.37–0.88; *p*< 0.011) in case of medium level exposure (40–80 pg/m^3^) and 0.49 (CI 0.29–0.8 1; *p*< 0.006) in case of high level exposure (>80 pg/m^3^) compared with the reference exposure (<40 pg/m^3^) [29]. Because in this study the exposure window was retrospectively defined with respect to the date of conception, Slama et al. reanalyzed the data after defining exposure window with respect to the start of unprotected intercourse and examined the effects of others air pollutants. Slama et al. did not confirm the effect of SO_2_ exposure before conception but they observed that each increase of 10 μg/m^3^ in PM2.5 concentration was associated with a 22% decrease in fecundability (95% CI = 6–35%). Among the other air pollutants studied (PAH, O_3_, NO_2_), only NO_2_ levels were significantly associated with a decreased fecundability in the first month (adjusted Fecundability Ratio (FR) = 0.71 [95% CI = 0.57–0.87]) and two first months (adjusted FR: 0.72 [95% CI: 0.53–0.97])of unprotected intercourse [30]. In Barcelona, using a cross-sectional study based on registry data at census tract level and a land use regression modeling approach, Nieuwenhuijsen et al. reported a statistically significant link (detailed in Table 1) between a decrease in the fertility rate (number of live births per 1000 women) and an increase in the level of air pollution, notably PM2.5–10 [31]. Lastly, in a recent study, Mahalingaiah et al. compared the risk of infertility in over 36,000 nurses as a function of their exposure to air pollution at their place of residence. In a multivariate analysis, they observed a significantly positive association between infertility and the proximity (<200 m) of the residence to a main road (Hazard Ratio [95% CI] for infertility when living closed to major roads compared to farther = 1.11 (CI: 1.02–1.20), and between infertility and the level of PM2.5–10. They therefore concluded that air pollution has a potentially harmful effect on fertility [32].

In conclusion, both animal and human data are not strong enough to point to a single air pollutant as responsible for a decrease in spontaneous fertility. Most of human data come from retrospective studies, based on declarative answers, predicted/modeled exposure levels and fail to take into account important confounders such as tobacco exposure. However, the only prospective human study based on a large population (36,294 women) and precise geolocalization data, found an impact of the proximity of residential address to major roads on the risk of infertility [32], which corroborates the results from mice studies [27, 28].

### **Impact of air pollution in vitro fertilization (IVF) outcomes** (Table 2)

Studying IVF populations helps provide evidence on the effects of air pollution on human reproduction since it allows to accurately time the key events in ovulation, fertilization and implantation. In this section, we excluded studies on the influence of air quality at IVF laboratories to concentrate on the effects of pollutants on patients undergoing IVF. The influence of environmental factors on assisted reproductive technology (ART) results, and notably on IVF techniques, has been suspected for many years [33], but the specific effects of air pollution on IVF have been little studied in the literature [26].

**Table 2:** Effect of pollutants on IVF outcomes (ICM: Inner cell mass; TE: trophectoderm)

Publication	Species	Number of subjects	Air pollutant (s) studied	Methodology	Results
Legro et al., 2010 [34]	Humans	7403 patients in first IVF cycle	PM2.5, PM10, SO_2_, NO_2_, O_3_	Retrospective cohort study Examined association between live birth rate and predicted daily pollutant exposure at the place of residence from first day of ovarian stimulation to day of oocyte retrieval (T1); from oocyte retrieval to embryo transfer (T2); from embryo transfer to pregnancy test (T3) and from embryo transfer to pregnancy outcome(T4)	Odd ratio (95%CI) for live birth associated with 1 SD increment of each pollutant: NO_2_ at T1: 0.80 (0.71–0.91) NO_2_ at T2: 0.87 (0.79–0.96) NO_2_ at T3: 0.76 (0.66–0.86) O_3_ at T1: 1.26 (1.10–1.44) O_3_ at T3:1.23 (1.07–1.41) O_3_ at T4: 0.62 (0.48–0.81)
Perin et al., 2010 [36]	Humans	531 pregnant women	PM10	Retrospective matched (study in infertile (*n* = 177) and spontaneously conceiving women (*n* = 354). Measured average exposure for 14 days following date of last menstrual period and association with first trimester pregnancy loss	Odd Ratio (95% CI) for first trimester miscarriage in fourth quartile of PM10 exposure level: -Total population: 2.58 (95% CI: 1.63–4.07); −in Infertile population: 2.32 (1.00–5.43) -Natural conception population: 2.72 (1.51–4.89).
Perin et al., 2010 [35]	Humans	400 women in first IVF cycle for male infertility	PM10	Retrospective study: measured average exposure during 14 days following the date of last menstrual period and association with laboratory outcomes (number of oocytes, fertilization rate, embryo morphology; *n* = 348), IVF treatment outcomes (*n* = 348) and pregnancy outcomes (*n* = 189).	No association between exposure to high concentrations of PM10 and laboratory and IVF treatment outcomes. Odd ratio (95% CI) for clinical early pregnancy loss in fourth quartile of PM10 exposure level: 5.05 (1.04–24.51).
Maluf et al., 2009 [37]	Mice	112	Multiple air pollutants from automobile traffic	3 groups of virgin females exposed to filtered (FA) or ambient air (AA) during prenatal (from date of efficient mating to delivery) and/or postnatal period (from delivery to 6 weeks of age): FA-FA group (*n* = 40); FA-AA group (*n* = 36) and AA-AA group (*n* = 36).	Significant effect of exposure to PM2.5 on blastocyst development.: ICM cell count decreased: FA-AA: 24.45 ± 5.58 AA-AA: 24.08 ± 4.79 FA-FA: 30.06 ± 6.32 TE cell count increased: FA-AA: 102.60 ± 10.82 AA-AA: 95.43 ± 12.28 FA-FA: 90.64 ± 10.11
Januario et al., 2010 [42]	Mice	225 zygotes (exposure 1) and 95 zygotes (exposure 2)	Diesel exhaust particles (DEP)	In vitro embryo culture with Exposure 1: 0, 0.2, 2 and 20 μg/cm^2^ DEP (10 μg/cm^2^ relevant to concentrations of ambient air) until day 5 Exposure 2: 0, 0.2, 2 and 20 μg/cm^2^ DEP until day 8	Exposure 1: ICM cells count decreased with increasing DEP concentrations: 0 μg/cm^2^:29.9 ± 2.5 0.2 μg/cm^2^:18.2 ± 3.5 2 μg/cm^2^:14.6 ± 6.5 20 μg/cm^2^:10.3 ± 4.1 TE cells count unchanged Exposure 2: Increased apoptotic cells at Day 8 with increasing DEP concentrations: 0 μg/cm^2^: 8.6% 0.2 μg/cm^2^:17.2% 2 μg/cm^2^: 22.1%

In 7403 women undergoing their first IVF cycle, Legro et al. assessed the effects of various air pollutants (SO_2_, NO_2_, O_3_, PM2.5 and PM10) along 4 different steps of the procedure: from the first day of ovarian stimulation to oocyte retrieval (T1); from oocyte retrieval to embryo transfer (T2); from embryo transfer to pregnancy test (T3) and from embryo transfer to pregnancy outcome (T4) [34]. They found negative impacts of a one standard deviation increase in NO_2_ concentrations on live births in all stages of the IVF cycle except T4 (Table 2). However this impact was stronger when the increase in NO_2_ concentrations occurred in T3 (Odd ratio (95%CI) for of live birth: 0.76 (0.66–0.86). Although, a biphasic effect of O_3_ exposure was seen, with a positive effect on live birth when exposure took place before embryo implantation and a negative effect after embryo implantation, no significant effects were observed in the live birth rate for other pollutants after adjusting for NO_2_ exposure.

Furthermore, in their study of the impact of short-term exposure (14 days after the date of the last menstrual period) to large particulate matter (PM10) on the results of IVF in about 400 women, Perin et al. did not observe any influence of exposure to PM10 on the ovarian stimulation parameters (number of days of treatment, ovarian response, etc.), on the biological parameters (number of oocytes gathered, fertilization rate, embryo morphology, etc.), or on the rates of embryo implantation and pregnancy. On the other hand, they found a statistically significant increase of 5% in the risk of early pregnancy loss per unit increase in follicular phase PM10 exposure, leading to an increased rate of early miscarriages among women exposed to the highest quartile of concentrations of PM10 [35]. In another study on 531 pregnant women, the same authors found that women exposed to high concentrations of large particulate matter (PM10) during the follicular phase of the ovarian cycle had a two-fold increased rate of early miscarriages, no matter if the conception was natural or the result of IVF [36].

Regarding animal data, Maluf et al. assessed the effects of exposure to fine particulate matter (PM2.5) from automobile traffic on the development of mouse embryos obtained through IVF from female mice exposed or not to PM2.5 during their pre- or postnatal period until sexual maturity [37]. They did not find any difference between exposed and unexposed mice in terms of the ovarian response to stimulation and the number of blastocysts obtained. However, they did observe a significant effect of PM2.5 exposure on the cell lineage allocation at the blastocyst stage between inner cell mass (ICM i.e. cells participating in the ontogeny of the future fetus) and trophectoderm (TE i.e. cells participating in the ontogeny of the future placenta). Indeed, although similar total blastocyst cells number were found, the number of cells in ICM were significantly increased in unexposed animals (30.06 ± 6.32) compared to pre and postnatally exposed (24.08 ± 4.79) or only postnatally exposed animals (24.45 ± 5.58). Oppositely, the number of cells in TE was decreased in blastocysts from unexposed animals leading to a weaker ICM/TE ratio in exposed animals by about 25%. It is well known in mice [38, 39], that a modified ICM/TE ratio impact on the blastocyst implantation potential and post-implantation outcome. In humans, although this ratio cannot be implemented in a clinical setting, the morphological grading of ICM and TE is linked with embryo ploidy [40] and impacts on blastocyst potential even in euploid blastocysts [41].

In another study, in vitro exposure of mice embryos to diesel exhaust particles extracted from the exhaust pipe of a bus from the Sao Paulo’s public transportation fleet, showed a negative dose-dependent effect on early embryo development and the hatching process, blastocyst cell allocation, ICM morphology and embryonic cells apoptosis [42].

Altogether, the data from the 3 available human studies about air quality and IVF results provide a weak level of evidence because they consist in retrospective studies, with long observation periods (7 [34] to 10 years [35, 36] during which effectiveness of IVF procedures may have improved), with approximated exposures based either on estimated levels from national models of air quality [34] or on average daily exposure of an entire city [35, 36], without accounting for the exact home address [35, 36] or its distance from the nearest monitoring station [34] or with residual confounding from tobacco exposure [34]. Furthermore, the results of these studies are discordant regarding PM10, the only air pollutant commonly evaluated by these studies. This could be due to large differences in PM10 levels between the study sites. Therefore other studies, ideally prospective, are needed to confirm the impact of air pollutants on human ART results.

### Impact of air pollution on the male gamete

#### In animals (Table 3)

Studies carried out on animals have found that various forms of air pollution have harmful effects on sperm quality. A statistically significant decrease in the daily production of spermatozoa has been reported along with an increase in abnormal sperm shapes in mice and rats exposed to car exhaust, notably from diesel vehicles [43–47]. An effect on the nuclear quality of spermatozoa has also been reported [48]. Yauk et al. observed a statistically significant increase in sperm DNA breakage and sperm DNA hypermethylation in mice exposed to ambient air pollution in a Canadian city [22]. These observations were associated with a statistically significant increase in the rate of mutations found in sperm DNA, especially on the loci of DNA sequence repeats. This last phenomenon raises the possibility of genetic mutations in the DNA of germline cells (spermatozoa in this case), that are transmissible to descendants [49].

**Table 3:** Effect of pollutants on spermatogenesis in animals

Publication	Species	Number of subjects	Air pollutant (s) studied	Methodology	Results
Ieradi et al., 1996 [45]	Mice	82	Multiple pollutants from automobile traffic	3 groups of mice at 3 sites in Rome exposed to varying traffic density. Samplings of epididymal sperm from males.	Increase in morphological abnormalities of spermatozoa in group exposed to highest levels of pollutants.
Watanabe and Oonuki, 1999 [47]	Rats	18	Multiple pollutants from diesel exhaust	3 groups of rats exposed from birth to age of 3 months: - group 1: exposed to diesel exhaust - group 2: exposed to filtered diesel exhaust (no particles) - group 3: unexposed control group	Increased levels of testosterone, estradiol and FSH in 2 exposed groups. Decreased level of LH in group 1. Decreased sperm production and testicular enzyme activity in 2 exposed groups.
Yoshida et al., 1999 [43]	Mice	80	Multiple pollutants from diesel exhaust	Male mice divided into 4 groups exposed for 6 months: - 3 groups with different concentrations of diesel exhaust particles (DEP): 0.3, 1.0 and 3.0 mg DEP/m^3^–1 unexposed control group	Altered morphology of seminiferous tubules, decreased mRNA in LH receptor and decreased daily production of spermatozoa in 3 exposed groups, in a non-statistically validated dose-dependent manner.
Tsukue et al., 2001 [51]	Rats	344	Multiple pollutants from diesel exhaust	Male mice divided into 4 groups exposed for 8 months: - 3 groups with increasing concentrations of diesel exhaust - 1 unexposed control group	Decreased prostate and coagulating gland weights and increased levels of LH and testosterone in group exposed to lowest concentration. Increased weight of prostate, seminal vesicles, and coagulating glands and testosterone levels in group exposed to highest concentration.
Somers et al., 2002 [48]	Mice	40 couples	Multiple pollutants from industrial sources	Mouse couples divided into 2 groups, exposed for 10 weeks: - 1 group in polluted industrial area - 1 unexposed comparison group in rural area Studied germline mutations (ESTR loci).	Increase in rate of germline mutations among mouse families in the exposed group. No difference in maternal mutations.
Inyang et al., 2003 [50]	Rats	40	Benzo (*a*) pyrene (BaP)	Males divided into 4 groups: - 3 groups exposed to increasing doses of BaP for 10 days - 1 unexposed control group	Decreased percentage of spermatozoa with progressive motility in 2 groups exposed to highest BaP concentrations
Somers et al., 2004 [49]	Mice	168	Multiple pollutants from industrial sources	4 groups of 21 outbred mouse couples: - 2 groups exposed in a polluted urban industrial area for 10 weeks: 1 group exposed to ambient air and 1 group exposed to air filtered with a HEPA filter (particle filter). - 2 groups exposed in a rural area for 10 weeks: 1 group exposed to ambient air and 1 group exposed to air filtered with a HEPA filter.	Exposure site and HEPA filtration both had significant impacts on paternal inherited mutations in offspring (ANOVA: F = 7.22, *p* = 0.009 and F = 8.03, *p* = 0.006 respectively). No effect on maternal mutations. Offspring of mice exposed to ambient air in urban industrial area inherited ESTR mutations of paternal origin 1.9 to 2.1 times as frequently as offspring in other three treatment groups.
Watanabe, 2005 [44]	Rats	156	Multiple pollutants from diesel exhaust	Pregnant females divided into 5 exposure groups, from 7th day of gestation to delivery: - group 1 exposed to high total dose of unfiltered diesel engine exhaust (high total) - group 2 exposed to high dose of filtered exhaust without particles (high filtered) _ group 3 exposed to low total dose of diesel engine exhaust (low-total) - group 4 exposed to low dose of filtered exhaust (low-filtered) - group 5 exposed to clean air (control) Study of males born of these groups.	Decreased number of Sertoli cells, number of germ cells and number of spermatozoa produced daily and increased follicle-stimulating hormone levels in all groups exposed to diesel exhaust
Jeng and Yu, 2008 [46]	Rats	20, 5 in each group	PAH	Males divided into 4 groups: - 3 groups exposed to increasing doses of PAHs - 1 unexposed control group	Lower daily sperm production and sperm mobility in exposed group. Increased LH levels and decreased testosterone levels in exposed group.
Yauk et al., 2008 [22]	Mice	30	Multiple pollutants from industrial sources and automobile traffic	Inbred males raised at polluted site and divided into 2 groups: - 1 group exposed to ambient air - 1 group exposed to air filtered with a particle filter (HEPA) Study of sperm DNA after 3, 10 or 16 weeks of exposure.	Increased frequency of ESTR locus mutations (after 16 weeks), number of DNA strand breaks (after 3 and 10 weeks) and increased DNA methylation (after 10 and 16 weeks) in group exposed to ambient air compared to filtered air.

On the testicular level, Yoshida et al. have observed structural changes in Leydig cells [43], while Watanabe has reported a reduction of Sertoli cells in rats exposed to diesel exhaust [44]. On the hormonal level, Jeng and Yu have demonstrated that extended exposure to PAHs leads to a reduction in blood testosterone levels and an increase in LH levels at the end of the exposure period [46]. Similarly, Inyang et al. found a statistically significant decrease in blood testosterone levels and an increase LH levels in rats exposed to benzo (a) pyrene, a type of PAH [50]. On the other hand, in their study on rats, Tsukue et al. described hormonal modifications in the group exposed to diesel exhaust with a statistically significant increase in blood testosterone levels and LH levels, associated with changes in the weight of the accessory sex glands (prostate, seminal vesicles) [51]. Watanabe and Oonuki also reported a statistically significant increase in the levels of estrogens and testosterone and a significant decrease in LH and FSH levels in a group of rats exposed to diesel exhaust. Furthermore, they observed an increase in the number of degenerative cells between the spermatocyte and spermatid stages [47].

#### In humans (Table 4)

Over the past few decades, a decline in the quality of sperm has been observed in industrialized countries [52, 53]. One possible reason for this alteration appears to be exposure to toxic substances in the environment, and notably ambient air pollution [54–56]. It has indeed been demonstrated that professions exposed to exhaust, such as toll collectors working on expressways, more frequently develop sperm abnormalities [16, 57].

**Table 4:** Effect of pollutants on spermatogenesis in humans

Publication	Number of subjects	Air pollutant (s) studied	Methodology	Results
Selevan et al., 2000 [59]	272	PM10, SO_2_, CO and NOx	Cross-sectional study: Compared sperm parameters of healthy 18-year-old men living in Teplice (industrialized area in Czech Republic) and those of healthy 18-year old men living in Prachatice (rural area in Czech Republic). Exposure data for PM10, SO_2_, CO and NOx gathered for 90 days before sperm sample taken and categorized in low, medium and high levels.	Men living in Teplice had significant decreased sperm mobility (mean ± SD: 31.6 ± 16.3 vs 36.1 ± 17.9), normal sperm morphology (16.6 ± 7.3 vs 19.3 ± 8.6). No effects seen on sperm count or sperm chromatin quality. Whatever the district, compared to low exposure level, medium exposure level was associated with decreased sperm motility (adjusted regression analysis: β (95CI): −8.03 (−13.57;-2.49)) and sperm morphology.(OR (95CI): −0.54 (−0.86;–0.22) High exposure level was associated with decreased normal sperm morphology (β(95CI): −0.84 (−1.15;–0.53)) and increased proportion of sperm with abnormal chromatin (β(95CI): 0.30 (0.08–0.52).
De Rosa et al., 2003 [54]	170	Multiple pollutants from automobile traffic	Cross sectional study: Compared exposed men (*n* = 85, working at an expressway toll plaza) to age-match control group (*n* = 85) from same area and employed as clerks, drivers, students or doctors.	In the exposed group compared to control group, Decreased sperm motility: 34.7 ± 2.2%vs 56.8 ± 0.8 (*p* < 0.0001) Decreased vitality: 51.7 ± 2.5% vs 80.7 ± 0.6 (*p* < 0.0001) Decreased sperm nuclear DNA integrity: 48.5 ± 2.2% vs 75.7 ± 0.6 (*p*< 0.0001) Decreased cervical mucus penetration: 15.9 ± 1.2 mm vs 30.3 ± 0.2 (*p* < 0.0001) No effect on sperm count and semen volume
Gaspari et al., 2003 [21]	182	PAH	Prospective cohort study: Study of sperm parameters in infertile Italian men with abnormal sperm morphologies who were partners of women without known causes of infertility. Measured sperm PAH-DNA adducts.	Positive correlation between level of sperm PAH-DNA adducts and abnormal morphology of sperm heads (*r* = 0.3; *p* = 0.0001).
Rubes et al., 2005 [68]	36	SO_2_, NOx and PM10	Prospective cohort study: young healthy men living in Teplice (industrialized area) who gave 3 to 7 sperm samples during winter (highly polluted air) and summer (less polluted air). Average concentrations of SO_2_, NOx and PM10 gathered in 90 days before each sampling.	Positive association between percentage of spermatozoa with abnormal chromatin and level of air pollution: β = 0.19 (95% CI: 0.02, 0.36) No association between exposure to air pollution and routine semen measures or sperm aneuploidy.
Hsu et al., 2006 [65]	48	PAHs	Cross sectional study: Semen evaluation among coke-oven workers at steel company in Taiwan. Compared “topside-oven” (TO, *n* = 16) most exposed group and “side-oven” least exposed group (SO, *n* = 32) group. PAH exposure measured in ambient air and urine.	Significantly higher rates of oligospermia (18.8 vs. 0%) and abnormal sperm morphology (32.3 vs. 14.6%) in TO vs. SO workers. No difference in semen volume, sperm count, motility, and frequency of asthenospermia. Positive correlations between urinary PAH level and percentage of abnormal sperm shapes (β (SE β): 0.107 (0.040); *p =* 0.012) and decondensed sperm chromatin (β (SE β): 0.235 (0.073); *p* = 0.003).
Sokol et al., 2006 [67]	48	O_3_, NO_2_, CO and PM10	Retrospective cohort study: Evaluated sperm count and motility from sperm donors (*n* = 48) who provided 5134 sperm donations over 2 year period. Exposure data for O_3_, NO_2_, CO and PM10 from donor’s place of residence over 3 periods: 0–9, 10–14, and 70–90 days before each donation.	Negative association between the level of O_3_ exposure and total sperm count: For 0–9 day lag: 4.22% decrease per interquartile range (IQR) of 14.3 ppb increase in O_3_, *p* = 0.01 For 10–14 day lag: 2.92% decrease per IQR of 14.3 ppb increase in O_3_, *p* = 0.05) For 70–90 day lag: 3.90% decrease per IQR of 14.3 ppb increase in O_3_, *p* = 0.05) No association between the level of O_3_ exposure and total motile sperm count. No association with other pollutants studied.
Guven et al., 2008 [57]	73	Multiple pollutants from diesel exhaust	Cross sectional study: Compared semen parameters of men exposed to diesel vehicle exhaust (*n* = 38 men working as toll collectors at motorways) to men working as office personnel in same company (*n* = 35). No monitoring of exposure levels.	Significant decrease in sperm concentration (mean ± SD: 44.64 ± 36.26 vs 70.85 ± 50.0), mobility (54.76 ± 23.64 vs 70.25 ± 15.5) and sperm with normal morphology (*p* = 0.001) in exposed group compared to unexposed group.
Hammoud et al., 2010 [58]	2576 samples	PM2.5	Retrospective study of 2 populations over 5 years: Population 1: men attending a semen analysis (*n* = 1699 samples, 1.16 ± 0.46 (mean ± SD) semen samples per patient, number of patient not stated) Population 2: men presenting for artificial intrauterine insemination on at least four occasions (*n* = 169 patients, 877 samples). Local average monthly concentrations of PM2.5 in each of 4 months preceding sampling, based on national data.	Population 1: Contemporaneous PM 2.5 correlated negatively with current sperm morphology (*r* = −0.076;*p* = 0.018) Negative correlation between PM 2.5 recorded 2 months (β = − 0.510; *p* = 0.01) and 3 months (β = − 0.411; *p* = 0.04) previously andsperm motility. Population 2: Sperm motility correlated negatively with PM 2.5 values recorded 3 months previously (β = − 0.407; *p* = 0.04) No correlation between semen parameters and PM 2.5 values recorded 1, 3, and 4 months previously in both populations.
Jurewicz et al., 2015 [70]	212	O_3,_ CO, SO_2_, NOx, PM2.5 and PM10	Prospective cohort study: Measured level of sperm aneuploidy in Polish men consulting for infertility with normal sperm counts. Exposure data for average CO, SO_2_, NOx, PM2.5 and PM10 over 90 days before sampling at closest station to place of residence.	Positive association between PM2.5 exposure and disomy Y (β = 0.68 (95%CI: 0.55–0.85); *p* = 0.001), sex chromosome disomy (β = 0.78 (95%CI: 0.59–0.99; *p* = 0.05), disomy of chromosome 21 (β = 0.78 (95%CI: 0.62–0.97; *p* = 0.03). Positive association between PM10 exposure and disomy 21 (β = 0.58 (95%CI: 0.46–0.72; *p* = 0.02) No association between sperm aneuploidy and O_3_, CO, SO_2_ and NOx exposures.
Wijesekara et al., 2015 [62]	300	Multiple pollutants from environmental and occupational exposures	Cross-sectional study: Male partners from infertile couples with no known male cause, divided into exposed and unexposed groups according to environmental and occupational exposure to pollutants based on interviewer- administered questionnaire.	Among normozoospermic patients (*n* = 201), exposed men (*n* = 115) showed, compared to non-exposed men (*n* = 86), significant decreases in (mean (95%CI): Normal morphology (%): 39.03 (36.25–41.81) vs 43.89 (40.54–47.24) Sperm mobility (%): 48.42 (46.57–50.27) vs 52.48 (50.2–54.76) Sperm vitality(%): 56.67 (54.48–58.86) vs 64.71 (62.03–67.39) Among pathozoospermic men (*n* = 99), only sperm concentration (16.89 million/ml (12.25–21.53) vs 31.94 (23.45–40.43)) and sperm morphology (normal forms: 21.46% (17.2–25.72) vs 28.00% (23.35–32.65)) were affected in exposed vs non-exposed men.
Radwan et al., 2016 [66]	327	PM10, PM2.5, SO2, NOX, CO	Retrospective cohort study: Polish men with normal sperm counts treated for infertility. Obtained exposure data for average CO, SO_2_, NOx, PM2.5 and PM10 over 90 days before sperm sampling at closest station to place of residence. Conducted multiple linear regression after adjusting for age, smoking, mean air temperature (over 90 days before sperm sampling), past diseases, duration of sexual abstinence and season.	Statistically significant associations between sperm morphology and exposure to each pollutant.: PM10: β =32.60; *p* = 0.0002 PM2.5: β =40.53; *p* = 0.0001 SO2: β =6.60; *p* = 0.0001 NOx: β =6.19; *p* = 0.01 CO: β =24.86; *p* = 0.0001. Statistically significant associations between the percentage of spermatozoa with immature chromatin and levels of PM2.5 (β =0.31; *p* = 0.0001) and PM10 (β =0.22; *p* = 0.02).

The literature on this subject is rich, but the existing studies are not always comparable because they do not necessarily concern the same pollutants and their methodologies often differ in terms of study populations, and duration and period of exposure. Moreover, their results are sometimes discordant.

Nonetheless, most of the studies find alterations in sperm parameters after exposure to air pollution, providing evidence for a decrease in sperm quality. These alterations involves a reduction in sperm mobility [54, 56–62], or in the quality of their movement [54, 56]. Altered sperm morphology with a reduction in the percentage of normal shapes, notably the morphology of the head, is also frequently mentioned [21, 56, 57, 59–66]. There are discordant results for sperm counts, with some studies reporting a significant decrease in the sperm concentration in semen after exposure to certain forms of air pollution [56, 57, 60, 65, 67], whereas others not observing any significant effects [59, 64, 68]. The same is true for proportion of alive spermatozoa (sperm vitality), with a small number of studies finding a significantly negative effect of air pollution on this parameter [54, 62].

In their study, Guven et al. compared sperm parameters among men exposed to exhaust from diesel vehicles through their work at toll plazas on expressways to unexposed men working as office personnel in the same company. The exposed group had a statistically significant decrease in sperm counts, sperm mobility and sperm morphology notably cephalic defects [57]. Selevan et al. studied sperm parameters in healthy young men from two regions of the Czech Republic, a coal-producing region with high levels of air pollution (Teplice) and a less-polluted region (Prachatice). Compared to low level of exposure, they found a statistically significant negative impact of exposure to medium and high levels of air pollution on the proportion of motile sperm (respectively for low, medium and high exposure: 36.2% ± 17; 27.9% ± 18.1 and 32.5% ± 13.2). The same was true for sperm morphology, notably sperm heads. On the other hand, compared to low level of exposure, they did not evidence an effect of medium or high exposure to air pollution on total sperm count (respectively: for low, medium and high exposure: 113.5 ± 130.7 million/ejaculate; 100.9 ± 97.6 and 129.1 ± 103.1) [59]. The same team found a statistically significant increase in the percentage of spermatozoa with abnormal chromatin (abnormalities in DNA compaction and fragmentation) in men exposed to high levels of air pollution in the Teplice district of Czech Republic [68]. These studies thus suggest that air pollution may alter sperm DNA [48, 69].

Alongside these observations, other authors also reported a possible effect of air pollution on the sperm genome at the chromosomal level [60, 70]. Thus, Jurewicz et al. [70] measured the aneuploidy rate in the sperm of Polish men with normal sperm concentrations (> or = 15 million/ml) consulting for infertility. After adjustment for 12 confounding factors such as age, smoking, alcohol consumption, season, past diseases, abstinence interval, distance from the monitoring station, they observed a significant association between the aneuploidy rate and exposure to certain air pollutants, notably Y-chromosome disomy and PM2.5 (β = 0.68 (95% CI: 0.55–0.85)), disomy 21 and PM10 (β = 0.58 (95% CI: 0.46–0.72)) and PM2.5 (β = 0.78 (95% CI: 0.62–0.97)).

Lastly, as is the case in animals, some authors reported a change in the circulating levels of hormones in the gonadal axis following exposure to air pollution. De Rosa et al. compared a group of exposed men working at a toll plaza on an expressway to an unexposed group working as clerks, drivers, students or doctors and living in the same geographical area. Along with an alteration of sperm parameters (other than ejaculate volume and sperm count), they observed a significantly higher level of FSH in the exposed group (mean ± SE: 4.1 ± 0.3 UI/l vs 3.2 ± 0.2; *p* < 0.05), although this remained within the normal value range [54]. Radwan et al. [66] also found a negative association between testosterone levels and exposure to certain air pollutants (PM10, PM2.5, CO and NOx).

The evidence for air pollution’s harmful effect on male reproductive parameters is therefore strong. It may also favor a decrease in male fertility. However, the vast majority of the human studies are retrospective. Our search found only one prospective study over a 2-year period in young, non-smoker healthy sperm donors from Los Angeles, California [67]. The population was quite small (*n* = 48) and only sperm concentration and motility were studied. However, each donor provided at least 10 times during the studied period. Only O_3_ exposure showed a significant impact on sperm parameters among the 4 air pollutants estimated at place of residence (O_3_, NO2, CO and PM10) after adjustment for numerous factors including abstinence period and the other air pollutants. For exposure up to 9 days before semen collection, there was a 4.22% decrease in sperm concentration per interquartile range (IQR) of increase in O_3_ (*p* = 0.01). For exposure 10–14 days and 70–90 days before ejaculation, there were 2.92% and 3.90% decreases in sperm concentration per IQR of increase in O_3_, respectively (*p* = 0.05 in both cases). More longitudinal studies are needed to confirm the negative impact of air pollution on human semen parameters.

### Impact of air pollution on the female gamete (Table 5)

Contrary to the male aspect, very few studies have been carried out on the impact of air pollution on female reproductive parameters in spontaneous fertility. This is likely explained by the difficulties involved in such studies. Indeed, it is easier to gather and study male gametes. A small number of authors have nonetheless looked into this subject.

**Table 5:** Effect of pollutants on ovarian functions in animals and humans

Publication	Species	Number of subjects	Air pollutant (s) studied	Methodology	Results
Ogliari et al., 2013 [71]	Mice	37	Multiple pollutants from diesel exhaust	4 groups of females: - group 1 = intrauterine and postnatal (60 days) exposure to filtered air - group 2 = intrauterine exposure to polluted air from diesel exhaust and postnatal exposure to filtered air - group 3 = intrauterine exposure to filtered air and postnatal to polluted air - group 4 = intrauterine and postnatal exposure to polluted air Morphometric analysis of ovaries to define relative area occupied by primordial, primary, secondary, and Graaf follicles.	Significant decreased proportional area occupied by primordial follicles in all exposed mice, whether in utero (*p* = 0.035), during postnatal period (*p* = 0.015) or both (*p* = 0 .004). Proportions of primary follicles (*p* = 0.04) and secondary follicles (*p* = 0.05) were only reduced in mice exposed in utero.
Thurston et al., 2000 [72]	Humans	3343	Benzene	Cross-sectional study: Used standardized questionnaire to measure length of menstrual cycles in women working in petrochemical industry and determined association with exposure to benzene based on self-reports.	After 7 years of work, increase risk of having abnormal menstrual cycle length (less than 21 or more than 35 days) with every 5 years of additional benzene exposure: Odds Ratio: 1.71 (95% CI:1.27–2.31).
Cho et al., 2001 [73]	Humans	1408	Multiple pollutants from occupational exposure to organic solvents (benzene, toluene, styrene, and/or xylene)	Cross sectional study: Measured length of menstrual cycles based on questionnaire administered by interviewer in group of women working in petrochemical industry and exposed to organic solvents compared to unexposed group (based on qualitative industrial hygiene assessment) working in same company.	Compared to unexposed group, odds ratio (95%CI) of oligomenorrhea (menstrual cycles exceeding 35 days) in group exposed to: styrene: 1.65 (1.05–2.55) xylene: 1.63 (1.04–2.53) benzene: 1.35 (0.90–2.00) toluene: 1.43 (0.93–2.17) all solvents: 1.76 (1.08–2.82)
Tomei et al., 2006 [74]	Humans	201	Multiple pollutants from automobile traffic	Prospective cohort study: Compared levels of blood 17β-estradiol in follicular, ovulatory and luteal phases of cycle in female police officers assigned to automobile traffic (*n* = 100) to levels in control group of female police officers assigned to indoor activities (*n* = 101). Groups matched on numerous criteria including day of cycle.	Significant decrease in mean (SD) level of estradiol in exposed group during follicular phase (50.4 (21.1) vs 118.5 (71.1) pg/ml; *p*< 0.001) and luteal phase (82.3 (33.0) vs 153.9 (57.3) pg/ml; *p*< 0.001), but not in ovulatory phase (150.9 (91.6) vs 193.5 (112.5) pg/ml; NS).

#### In animals

Veras et al. compared a group of mice exposed to air polluted by automobile traffic in Sao Paulo to a group of mice exposed to less polluted filtered air. The authors observed a significant lengthening of the cycles as well as a decrease in the number of antral follicles in the exposed vs. unexposed groups, but they did not observe a significant effect on follicles at other stages of follicular development (i.e primordial, primary follicles and secondary follicles) [28].They therefore concluded that air pollution has a potentially toxic effect on ovary. Such effect was demonstrated later by the same group comparing the ovarian histology of female mice exposed to diesel exhaust (notably PM2.5) in utero and/or during the postnatal period to unexposed mice [71]. While exposure levels were considered acceptable by the World Health Organization, ovaries showed a statistically significant decrease in the proportion of the area occupied by primordial follicles in exposed mice in all exposure periods (prenatal exposure (from day of vaginal plug to birth); postnatal exposure (from birth until sexual maturity defined as post-natal day 60) or both) compared to unexposed mice. Thus, the authors concluded that there may be a possible decrease in ovarian reserve and therefore in the reproductive potential of mice following exposure to diesel exhaust, notably PM2.5 [71]. However, while the investigators planned to maintain PM2.5 exposure levels within acceptable daily ranges as defined by the World Health Organization (25 μg/m^3^/d), the daily average exposure was obtained from a 1 h exposure to a much higher level (660 μg/m^3^). Such an acute exposure could drastically impact the results.

#### In humans

Only three studies have been carried out among women. Two cross sectional studies have observed the length of menstrual cycles in large populations of women who worked in the petrochemical industry in China and were exposed to organic solvents. Thurston et al. studied the effect of an occupational exposure to benzene (a highly volatile monocyclic aromatic hydrocarbon) evaluated by self-report. They found that the adjusted odds of menstrual cycle abnormalities (fewer than 21 or more than 35 days) did not change significantly during the first 7 years of exposure. However after 7 years, the odds ratio increased to 1.71 (95% CI 1.27–2.31) per additional 5 years of exposure [72]. Cho et al. used a more objective exposure evaluation based on an industrial hygiene evaluation classifying each workplace according to the presence or absence of four organic solvents (benzene, toluene, styrene, and/or xylene) using the information on production steps in the processing of petrochemicals. They described a higher frequency of women with oligomenorrhea (menstrual cycles exceeding 35 days) in the group exposed to styrene, xylene or all solvents. Furthermore, one additional year of exposure to any solvent significantly increased the odds ratio by 7% (CI95%: 1.00–1.14) and a duration of work greater than 3 years yielded an adjusted odds ratio for oligomenorrhea of 1.53 (95% CI:1.00–2.34) [73]. Therefore, these two studies argue for an exposure duration-response relationship for aromatic solvents.

Tomei et al. studied the impact of exposure to pollutants on female police officers assigned to traffic control in Rome compared to a matched-control group of female police officers assigned to indoor activities such as administrative and bureaucratic duties. They found an average level of estradiol that was statistically significantly lower in the exposed group in the follicular phase and luteal phase of the cycle, but not in the ovulatory phase. Although no statistically significant difference between the two groups was noted in terms of disruption of the menstrual cycle, the authors suggest that these hormonal changes could alter ovulation in exposed women [74].

Although these three studies suggest that air pollution may have an impact on female reproductive parameters, notably at the ovarian level, questions remain about whether the pollutants have a direct or indirect effect on the hypothalamic-pituitary-gonadal regulation.

### Mechanisms of action of air pollutants

Four possible mechanisms have been put forward in the literature for the mechanism of action of air pollutants on fertility: hormonal changes due to an endocrine disruptor action, oxidative stress induction, cell DNA alteration or epigenetic modifications. Air pollutants can act as endocrine disruptors mainly through activation of the aryl hydrocarbon receptor (AhR) or estrogen or androgen receptors [75]. Another common cellular mechanism by which most air pollutants exert their adverse effects is their ability to act directly as prooxidants of lipids and proteins or as free radicals generators, promoting oxidative stress and the induction of inflammatory responses [76]. Some pollutants can alter the DNA molecule or induce epigenetic changes, such as DNA methylation and histone modifications which can be transmitted to future generations.

### Action as endocrine disruptors

Air pollutants notably the PAHs and heavy metals (such as Cu, Pb, Zn, etc.) contained in PM, especially from diesel exhaust [12, 13], are described in the literature as endocrine disruptors with either estrogenic, anti-estrogenic or anti-androgenic activity [14, 77–81].

Kizu et al. described anti-androgenic activity of certain particle compounds from diesel exhaust in human PC3/AR cells derived from prostate cancer tumors [15], while Okamura et al. reported anti-estrogenic activity of similar compounds in human MCF-7 cells from breast cancer tumors [14]. In vivo studies carried out on rats found significant changes in the circulating levels of sex steroids and gonadotrophins in groups exposed to diesel exhaust, associated with a decrease in the daily production of sperm, demonstrating inhibited spermatogenesis, as well as morphological changes to germ cells in the seminiferous tubules [46, 47, 50, 51]. Likewise, in their study on mice, Yoshida et al. reported a significant decrease in the level of messenger RNA expression in LH receptors of Leydig cells, and altered morphology of the seminiferous tubules and Leydig cells in the group exposed to diesel exhaust [43].

In men, a small number of studies describe a change in the circulating levels of hormones in the gonadal axis following exposure to air pollution. For example, De Rosa et al. found an alteration in sperm parameters associated with significantly higher levels of FSH in the group exposed to exhaust, although they remained within the normal value range [54], while Radwan et al. reported a negative association between testosterone levels and exposure to certain air pollutants (PM10, PM2.5, CO and NO_2_) [66].

These hormone disruptions may be induced by air pollutants, notably PAHs, binding to estrogen receptors [78, 79, 82, 83] or androgen receptors [15, 84] with an agonistic or antagonistic effect. They may also be the result of activation of AhR pathway. This is a transcription factor activated by a large number of ligands, including PAHs [82, 83, 85, 86] and involved in many cell processes. For example, extractable organic matter (EOM) from PM2.5 may cause heart malformations and decreased heart rates in zebrafish embryos through the AhR, and these heart defects appear to be counteracted in embryos co-exposed to EOM and an AhR antagonist [87, 88]. Concerning the male reproductive system, Izawa et al. have reported a decrease in the daily production of spermatozoa, an increase in morphological abnormalities in spermatozoa as well as a significant increase in blood testosterone levels in the group exposed to diesel exhaust combined with a significant increase in the AhR activity index [89]. Using strains of mouse with different AhR activity index, they showed that the decrease in daily sperm production due to diesel exhaust exposure was negatively correlated with this index (*r* = −0.593; *p* = 0.008) [90]. Concerning the female reproductive system, AhR may regulate ovarian follicle growth, modulate ovarian steroidogenesis and play a role in ovulation [91]. It also appears to relay the toxicity of certain ligands, such as PAHs, notably in the reproductive system, where it may be one of the mediators in steroid hormone disruption and therefore in fertility [89, 90, 92].

### Induction of reactive oxygen species (ROS)

Most air pollutants such as NO_2_are ROSor are capable of generating them, such as O_3_ or PM, through the heavy metals and the PAHs they contain. They can be transformed by CYP450 dihydro-dehydrogenase, which produces quinone redox, catalyzing electron transfer reactions and thus stimulating ROS production [93, 16–18].

For males, several studies have described the potentially harmful effects of oxidative stress on sperm. While a certain amount of ROS is needed for the physiological functions of spermatozoa, notably for the fertilization process, an excess amount may cause damage to the spermatozoa [93]. Indeed, the sperm membrane comprises a large number of polyunsaturated fatty acids that maintain the membrane’s fluidity, making sperm highly sensitive to oxidative stress. Peroxidation of these fatty acids can cause a loss of this fluidity as well as a decrease in activity of the membrane enzymes and the ion channels, and can thus alter sperm mobility and some of the mechanisms needed for fertilizing the oocyte. Furthermore, peroxidation of the sperm DNA bases could lead to breakage of the DNA strands and genetic mutations, causing a decrease in the sperm’s fertilization potential, along with an alteration of subsequent embryo development. The protein oxidation induced by ROS may also alter sperm functions by splitting the polypeptide chains and an accumulation of protein aggregates. Lastly, ROS may initiate chain reactions leading to apoptosis, notably by altering mitochondrial membrane integrity. This process could be sped up by the damage to the DNA and the sperm membrane, possibly leading to a decreased sperm count [94–98].

For women, ROS are produced during folliculogenesis and they also appear to play a physiological role, notably in renewed oocyte meiosis I and in inducing ovulation [99, 100]. Excess ROS, however, leads to a state of oxidative stress and appears to be harmful to ovarian functions. In their study of mutated mice with a deficit of glutathione, the most abundant intracellular antioxidant, Lim et al. described a faster decrease in the number of ovarian follicles in these mutated mice, related to an acceleration in primordial follicle depletion [101]. They also reported a higher percentage of small follicles with a heavy proliferation of granulosa cells, reflecting the accelerated recruitment of primordial follicles. They therefore concluded that oxidative stress in the ovaries may lead to a decrease in the ovarian reserve by speeding up the depletion of primordial ovarian follicles by increasing recruitment from the follicle pool and apoptosis at the most advanced stages of follicle development.

### Cell DNA alteration

The third mechanism reported in the literature to explain the pathophysiologic mechanisms involved in fertility alteration caused by air pollution is the induction of alterations in the cell DNA.

First, these DNA alterations could be linked to induced oxidative stress, as described above. Indeed, the inflammation processes due to ROS can alter DNA as reported in a study of taxi drivers [102]. Moreover, telomere length has been reported to increase with increasing annual exposure to NO_x,_ PM_2.5_ and PM_10_ [103].

Second, they may occur after the formation of DNA adducts. Indeed, some molecules are able to bind to a DNA base through covalent bonding, thus modifying gene expression. In addition, mutations may occur, leading to an alteration of the cell DNA and increased risk of apoptosis. Some air pollutants are capable of forming DNA adducts in germ cells, notably the PAHs contained in PM [21, 46, 68].

### Epigenetic modifications

Epigenetic modifications, notably changes in DNA methylation, can lead to abnormal gene expression. These abnormalities have been implicated in the effect of air pollution on carcinogenesis [104] and respiratory failure [105, 106]. In rats exposed to PM2.5, PM10 and NO_2_, Ding et al. have demonstrated both hypomethylation and hypermethylation of certain genes [107]. These changes can also affect mitochondrial (mt) DNA [108]. Byun et al. have shown that blood mtDNA methylation in the D-loop promoter was associated with PM2.5 levels [109] Epigenetic alterations have been also reported as involved in the failure of spermatogenesis [110]. In the study of Stouder et al. [111] alcohol administration in pregnant mice induced hypomethylation of H19 imprinted gene and may have contributed to the decreased spermatogenesis. In another study [112], Park et al. have shown that long-term exposure to butyl paraben (BP) can cause DNA hypermethylation from the mitotic through post-meiotic stage in adult rat testes.

Lastly, air pollutants have also been shown to alter microRNA (miRNA). A study by Tsamou et al. has shown that the placental expression of miR-21, miR-146a and miR-222, three miRNA known to be expressed in the placenta and to be affected by air pollution exposure in leucocyte blood cells, was inversely associated with PM_2.5_ exposure during the 2nd trimester of pregnancy [113].

## Conclusion

Air pollution has a negative impact on both male and female gametogenesis. These impacts not only influence the quantity of gametes but also on their quality on a genetic and epigenetic level. These impacts also alter the embryo development.

Most prior studies concern male gametes, probably due to the ease of obtaining and analyzing them, and few prior studies have focused on oocytes and folliculogenesis. Studies have also shown an impact on fetal development, with an increase in miscarriages.

The individual role of specific pollutants is difficult to identify, since subjects in the epidemiological studies are typically exposed to several pollutants simultaneously.

The physiopathology leading to altered fertility is poorly understood. Hormonal disturbances, oxidative stress induction, cell DNA and epigenetic alterations are four mechanisms put forward in the literature, probably working in combination, to explain this negative impact.

As air pollution is ubiquitous and has many origins, it would seem to be indispensable to increase awareness among the population and public authorities to attempt to limit air pollutants as much as possible.

## Abbreviations

AhR: aryl hydrocarbon receptor
ART: assisted reproductive technologies
DNA: deoxyribonucleic acid
EOM: extractable organic matter
FSH: follicle stimulating hormone
IVF: in vitro fertilization
LH: luteinizing hormone
miRNAs: micro ribonucleic acid
NO_2_: nitrogen dioxide
NOx: nitrogen oxides
O_3_: ozone
PAH: polycyclic aromatic hydrocarbons
PM: particulate matter
ROS: reactive oxygen species
